# Ambient Air Temperature Assisted Crystallization for Inorganic CsPbI_2_Br Perovskite Solar Cells

**DOI:** 10.3390/molecules26113398

**Published:** 2021-06-03

**Authors:** Yi Long, Kun Liu, Yongli Zhang, Wenzhe Li

**Affiliations:** 1 Institute of New Energy Technology, Department of Electronic Science and Engineering, College of Infomation Science and Technology, Jinan University, Guangzhou 510632, China; jianyidelong@163.com (Y.L.); kunliu2021@163.com (K.L.); 2 Department of Ecology, College of Life Science and Technology, Jinan University, Guangzhou 510632, China

**Keywords:** CsPbI_2_Br, perovskite solar cells, ambient air temperature assisted crystallization, film quality, photophysical properties

## Abstract

Inorganic cesium lead halide perovskites, as alternative light absorbers for organic–inorganic hybrid perovskite solar cells, have attracted more and more attention due to their superb thermal stability for photovoltaic applications. However, the humid air instability of CsPbI_2_Br perovskite solar cells (PSCs) hinders their further development. The optoelectronic properties of CsPbI_2_Br films are closely related to the quality of films, so preparing high-quality perovskite films is crucial for fabricating high-performance PSCs. For the first time, we demonstrate that the regulation of ambient temperature of the dry air in the glovebox is able to control the growth of CsPbI_2_Br crystals and further optimize the morphology of CsPbI_2_Br film. Through controlling the ambient air temperature assisted crystallization, high-quality CsPbI_2_Br films are obtained, with advantages such as larger crystalline grains, negligible crystal boundaries, absence of pinholes, lower defect density, and faster carrier mobility. Accordingly, the PSCs based on as-prepared CsPbI_2_Br film achieve a power conversion efficiency of 15.5% (the maximum stabilized power output of 15.02%). Moreover, the optimized CsPbI_2_Br films show excellent robustness against moisture and oxygen and maintain the photovoltaic dark phase after 3 h aging in an air atmosphere at room temperature and 35% relative humidity (R.H.). In comparison, the pristine films are completely converted to the yellow phase in 1.5 h.

## 1. Introduction

Since 2009, tremendous progress has been made in the power conversion efficiencies (PCEs) of organic–inorganic hybrid perovskite solar cells (PSCs), which have increased from 3.8 to 25.5% [1,2,3,4,5,6,7,8,9,10]. However, high temperature induces evaporation of the small organic components, e.g., methylammonium (MA), which could impede their commercialization [11,12]. In recent years, inorganic cesium lead halide perovskites (CsPbX_3_, X = Cl, Br, I) have attracted considerable attention due to their high-temperature stability, where a stable phase can be retained even at temperatures exceeding 400 °C [13,14,15]. Therefore, replacing the organic component with an inorganic component is widely regarded as the ultimate solution for addressing the thermal instability issue.

CsPbBr_3_ exhibits good stability but has a large bandgap (2.30 eV) [16]. However, according to the Shockley–Queisser limit, a wide bandgap dramatically limits its absorption and further photovoltaic performance improvement [17,18]. CsPbI_3_ displays a relatively narrow bandgap (1.73 eV) but suffers from notable phase instability [19]. CsPbI_2_Br is a novel inorganic halide perovskite with a mid-range bandgap (1.92 eV), and it can operate even in the ambient atmosphere [20]. However, the efficiencies of CsPbI_2_Br solar cells achieved to date are still low, principally as the result of poor CsPbI_2_Br film quality, which induces a low open-circuit voltage and poor moisture stability. Therefore, it is very desirable to control the crystallization process of CsPbI_2_Br for high-quality perovskite films.

It is acknowledged that obtaining high-quality perovskite films is a prerequisite to making high-efficiency and stable PSCs. Therefore, various fabrication methodologies, such as one-step-coating deposition [21], sequential deposition [22], spray-assisted solution-process [23], and vacuum evaporation [24], have been developed to produce high-quality CsPbI_2_Br films. Among these methodologies, the one-step-coating deposition method is highly desired for the further development of inorganic PSCs due to its simplicity, low cost, and effectiveness. Furthermore, various precursor solution engineering technologies have been developed to control the crystallization process to produce high-quality perovskite films, such as the Lewis acid–base adduct approach [25,26], temperature assisted crystallization of precursor solution [27], and antisolvent assisted crystallization [28]. However, these works are not related to the solvent atmosphere, humidity, and temperature of the spin-coating atmosphere. In order to obtain high-quality inorganic perovskite films, the spin-coating atmosphere is one of the key influencing factors in the perovskite film preparation.

Herein, we demonstrate that the film formation dynamics are controlled by adjusting the spin-coating temperature to 35 °C, which is beneficial to the highly crystalline and pinhole-free CsPbI_2_Br films, thereby yielding a remarkable enhancement of the ambient stability. We fabricated the mesoporous n-i-p type PSCs by using as-prepared CsPbI_2_Br film as a photoactive layer and achieved a PCE of 15.5% (the maximum stable power output of 15.0%). The optimized CsPbI_2_Br films show excellent moisture and oxygen stability and maintain a dark photovoltaic phase for 3 h in an air atmosphere with 35% relative humidity (R.H.). Correspondingly, the primitive film was completely converted to the yellow phase (δ-phase) in only 1.5 h. This work provides a new method for the preparation of highly efficient and stable inorganic perovskite PSCs.

## 2. Results and Discussion

**Figure 1:**
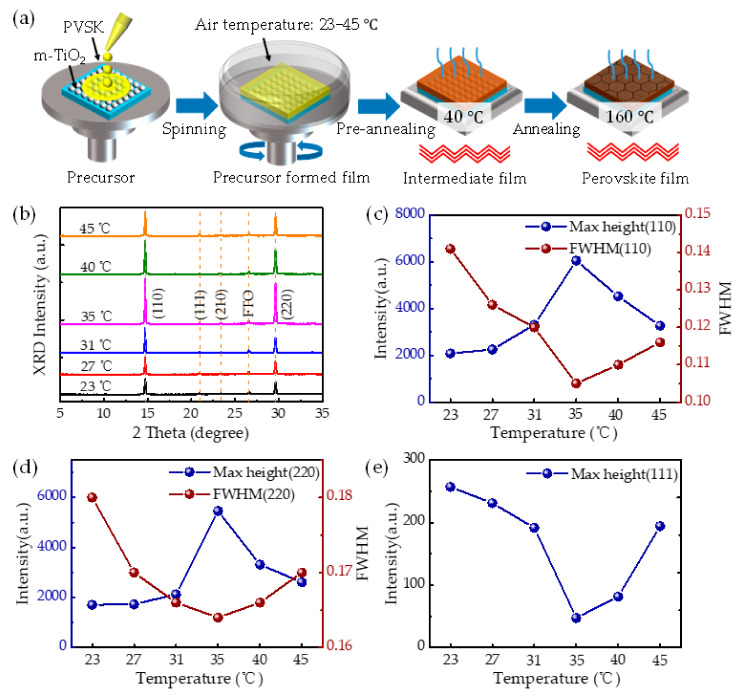
(**a**) Ambient air temperature assisted crystallization procedure of perovskite films. (**b**) X-ray diffraction patterns of the as-prepared films at different ambient temperatures. (**c**–**e**) Intensity and FWHM evolution of (110), (220), and (111) planes of perovskite films as the function of the ambient temperature.

We systematically explored the effects of spin-coating temperature on the structure of CsPbI_2_Br films. A temperature control system was embedded in the glove box to control the spin coating temperature. The route for fabricating the perovskite films is schematically shown in Figure 1a. The perovskite precursor was spin-coated on TiO_2_-coated FTO substrates at 3000 rpm for 50 s at different temperatures. CsPbI_2_Br film was immediately transferred to a 40 °C hotplate and kept for 1–4 min. The color of the intermediate film turned to brown. After the second step of annealing at 160 °C, X-ray diffraction patterns of the perovskite films were obtained (shown in Figure 1b). The diffraction peak of fluorine-doped tin oxide (FTO) glass at 26.2° was used as a reference to correct all the patterns. The XRD patterns show that all the films are β-phase with four main peaks at 14.7, 20.9, 23.4 and 29.5° assigned to the (110), (111), (210), and (220) planes of the tetragonal phase, respectively [29,30]. Figure 1c–e show the evolution of XRD peak intensity and full width at half maximum (FWHM) of perovskite films prepared at different ambient temperatures. The following phenomena can be observed: (i) The diffraction intensity of (110) and (220) planes of perovskite increase significantly to the maximum value at 35 °C, whereas the peak intensity of (111) plane tends to decrease. Air temperature assisted crystallization obviously improves the crystallized quality of perovskites along the (110) and (220) planes in this case. (ii) The crystallite size is inversely proportional to the FWHM of the diffraction peak according to the Scherrer formula in X-ray physics as follows [31]:

(1) $$D=\frac{K\lambda }{\beta cos\theta }$$

where D is the particle size in Å, K is the shape factor ≃0.9, λ is the wavelength (1.5418 Å, CuKα), β is the full width at half maximum (FWHM) of the peak, and θ is the peak position. Comparing the FWHM of (110) and (220) peaks, it can be found that the 35 °C sample has the smallest FWHM values, meaning the largest crystal size. The qualitative changes in the solid-state microstructural outcome point to the critical role of the film formation dynamics. Superior film quality can be achieved at the intermediate temperature of 35 °C. Temperatures lower than 35 °C would lead to a longer duration for turning to the brown intermediate phase, as was the case under 23–31 °C, which provides more opportunity for solvent gas adsorption. In the pre-heating process, the volatile rate of solvent is very crucial for the final quality of the perovskite films. Increasing the ambient temperature up to 35 °C can be an effective way to counterbalance the solvent gas attack on perovskite films, that is equally to use a pre-annealing step of 35 °C for 50 s without increasing the time of exposure to the gas solvent. It has been reported that the brown intermediate phase of CsPbI_2_Br can be obtained by pre-annealing at 35 °C, which has been used to prepare high-performance devices [32]. Hence, the largest crystalline grain obtained might be caused by low-temperature pre-annealing (35 °C), which induces slow formation of nuclei and growth of large crystalline grains. When the ambient temperature was increased up to 40–45 °C, the solvent volatilization rate was so fast that too many nuclei formed, which resulted in the reduced crystal size.

**Figure 2:**
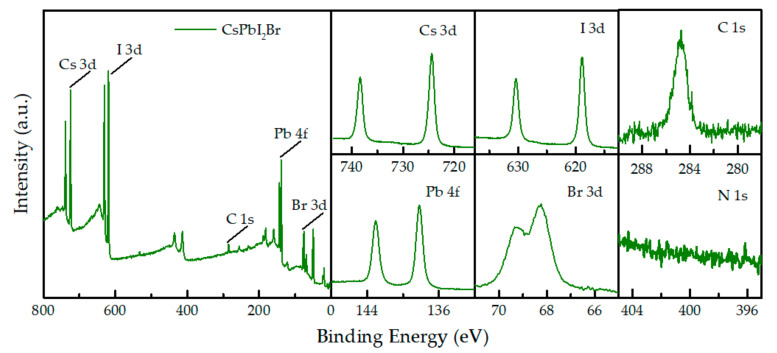
X-ray photoelectron spectroscopy of the perovskite films prepared at 35 °C in ambient atmosphere.

The elements and surface chemical states of halide perovskites were characterized by X-ray photoelectron spectroscopy (XPS) (Figure 2). The I 3d spectra exhibit two contributions, 3d 5/2 and 3d 3/2, located at 618.8 and 630.5 eV for the film prepared at 35 °C. The Br 3d spectra exhibit two peaks at 68.3 and 69.3 eV, respectively. The characteristic I4d peaks are located at 618.9 and 630 eV, and the Cs 3d peaks are located at 727.4 and 738.4 eV. The XPS spectra further confirm that the perovskite fabricated by ambient air temperature assisted crystallization is CsPbI_2_Br [32,33].

**Figure 3:**
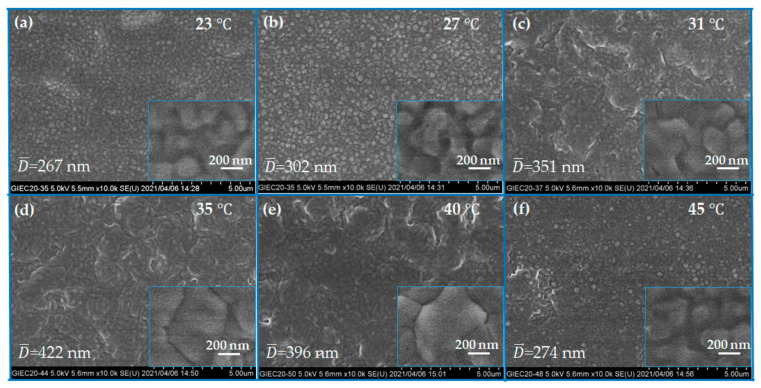
SEM images of CsPbI_2_Br perovskite films spin-coated at different ambient temperatures: (**a**) 23 °C; (**b**) 27 °C; (**c**) 31 °C; (**d**) 35 °C; (**e**) 40 °C; (**f**) 45 °C. $\bar{D}$ displays the average grain size of the corresponding perovskite thin films.

To elucidate the effect of ambient temperature on the morphologies of CsPbI_2_Br films, we employed SEM to investigate the evolution of their microstructure. The average grain ($\bar{D}$) was determined from the SEM images using an image processing and analysis software (ImageJ). The surface of each grain was measured, and its size was calculated in terms of the average of the longest and shortest diameter; the average grain sizes were determined from the measurement of 250 grains per sample [34]. As shown in Figure 3a–f, the results exhibit that the average crystal sizes gradually grow from 267 to 422 nm with the increase in temperature from 23 to 35 °C. When the temperature was increased to 45 °C, the average crystal size dropped quickly to 272 nm. The perovskite thin films were demonstrated to have various grain sizes [35,36], which were associated with different crystal growth dynamics and growth techniques. The effect of grain size on photovoltaic performance was studied. Remarkably, at the optimized temperature, i.e., 35 °C, the pinholes on the CsPbI_2_Br film surface became shallow or even disappeared; in the meantime, the shape of the grains changed from irregular to regular, which would give rise to good contact with the upper hole-transporting layer. This phenomenon is likely associated with the enhanced crystallinity of the CsPbI_2_Br films through ambient air temperature assisted process.

**Figure 4:**
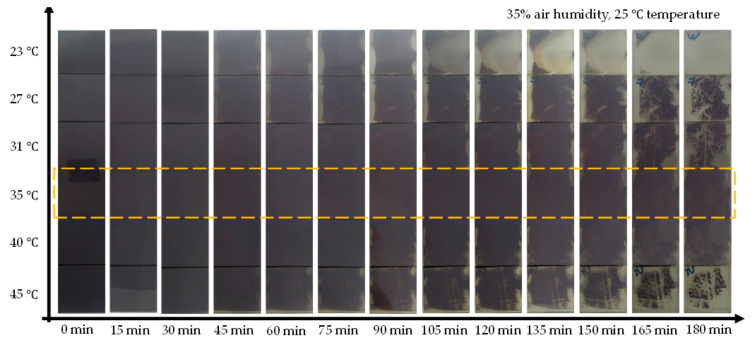
The morphology photos of CsPbI_2_Br perovskite thin films aged 180 min in air atmosphere with relative humidity (R.H.) of ~35%.

To clarify the stability of the above perovskite films, the long-term stability of unencapsulated CsPbI_2_Br films was tested in an air atmosphere with relative humidity (R.H.) of ~35%. As shown in Figure 4, where the moisture stability is strictly related to the processing temperature, it is obvious that the perovskite film processed at 35 °C shows higher stability. The improved stability of the perovskite films is ascribed to the better crystallinity of the perovskite films.

**Figure 5:**
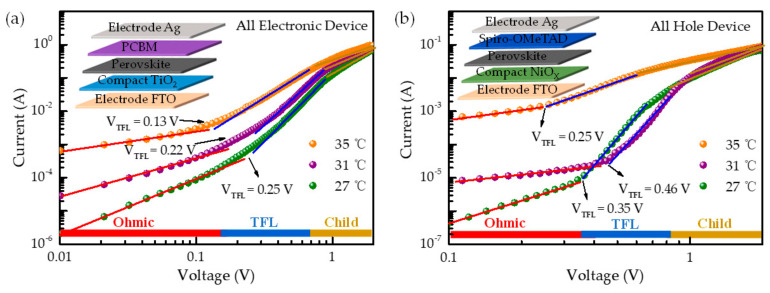
Dark *J–V* measurement of (**a**) the electron-only and (**b**) the hole-only devices with the perovskite films prepared at different ambient temperatures (inset shows the device structure).

In order to further verify the trap densities and carrier mobilities of the perovskite films, the dark *I−V* characteristics for electron-only devices and hole-only devices were obtained. The architectures of the devices were FTO/NiO/perovskite/phenyl-C61-butyric acid methyl ester (PCBM)/Ag and FTO/c-TiO_2_/perovskite/2,2′,7,7′-tetrakis (*N,N*-di-pmethoxyphenylamine)-9,9′-spirobifluorene) (Spiro-OMeTAD)/Ag. The curves for the 27 °C, 31 °C, and 35 °C films are presented in Figure 5. The trap density was determined by using the following equation [37]:

(2) $$N_{trap}=\frac{V_{TFL}\left(2\varepsilon_{r}\varepsilon_{0}\right)}{ed^{2}}$$

where $\;V_{TFL}$ is the onset voltage of the trap-filled limit region, $\varepsilon_{0}$ is the vacuum permittivity,$\;\varepsilon_{r}\;$ is the relative dielectric constant, d is the film thickness, and e is the elementary charge. The electron mobility was further extracted using the Mott–Gurney Law [38]:

(3) $$\mu =\frac{8J_{D}d^{3}}{9\varepsilon_{r}\varepsilon_{0}V^{2}}$$

where V is the applied voltage and $J_{D}$ is the current density. The electron trap density and electron mobility were estimated to be 2.5 × 10^14^ cm^−3^ and 4.8 × 10^−2^ cm^2^ V^−1^ s^−1^ for the 35 °C film, respectively (Figure 5a). By contrast, the electron trap density and the electron mobility of the other films (23–31°C is traditionally used) were found to have deteriorated. For example, the electron mobility was reduced to 6.0 × 10^−3^ cm^2^ V^−1^ s^−1^ and electron trap density increased to 4.2 × 10^14^ cm^−3^ for the 31 °C film; the electron mobility was reduced to 2.1 × 10^−3^ cm^2^ V^−1^ s^−1^ and the electron trap density increased to 4.8 × 10^14^ cm^−3^ for the 23 °C film. Likewise, the hole trap densities and the hole mobility were respectively calculated to be 4.7 × 10^14^ cm^−3^ and 6.1 × 10^−3^ cm^2^ V^−1^ s^−1^ for the 35 °C CsPbI_2_Br films, which were better than those of the other conditions (27 and 31 °C). It is worth noting that the 35 °C ambient temperature is beneficial in decreasing the defect density and thereby gives rise to superior transport properties, which is in agreement with the XRD and morphological characterizations. The low defect density and superior charge transport are expected to yield less charge recombination and higher power conversion efficiency in complete solar cells.

**Figure 6:**
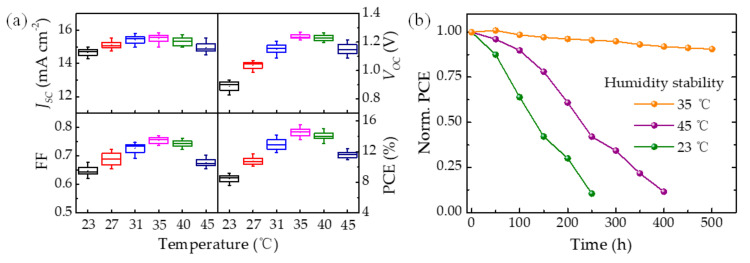
(**a**) Statistics on the photovoltaic parameters of perovskite solar cells. (**b**) Evolution of power conversion efficiency of the 23 °C, 35 °C, and 45 °C PSCs without encapsulation. The devices were stored under air ambient atmosphere with controlled relative humidity (R.H., 15 ± 3%).

We then carefully studied the photovoltaic performance of the as-fabricated perovskite solar cells prepared by tuning the ambient temperature of the spin-coating process (Figure 6a). Twelve devices in each condition group were used for data statistics. These data statistics reflect the reliability of device performance. Noticeably, the 35 °C device performed much better than others, especially in terms of the enhancement of open-circuit voltage (*V_OC_*) and fill factor (FF), which are associated with higher film quality (e.g., high crystallinity, good interface contact) [39,40,41]. Furthermore, the long-term stabilities of solar cell devices were tested under a relative humidity (R.H.) of ~15% by adopting the device configuration of FTO/c-TiO_2_/m-TiO_2_/perovskite/Spiro-OMeTAD/Au. As shown in Figure 6b, 10 devices in each condition group were used for data statistics. The results exhibit that the humid stability of the 35 °C device was noticeably enhanced, maintaining over 90% of its original PCE under ambient conditions with 15 ± 3% humidity for 500 h. In striking contrast, the 35 and 45 °C devices only display below 15% of their initial efficiency for 400 h under the same conditions.

**Figure 7:**
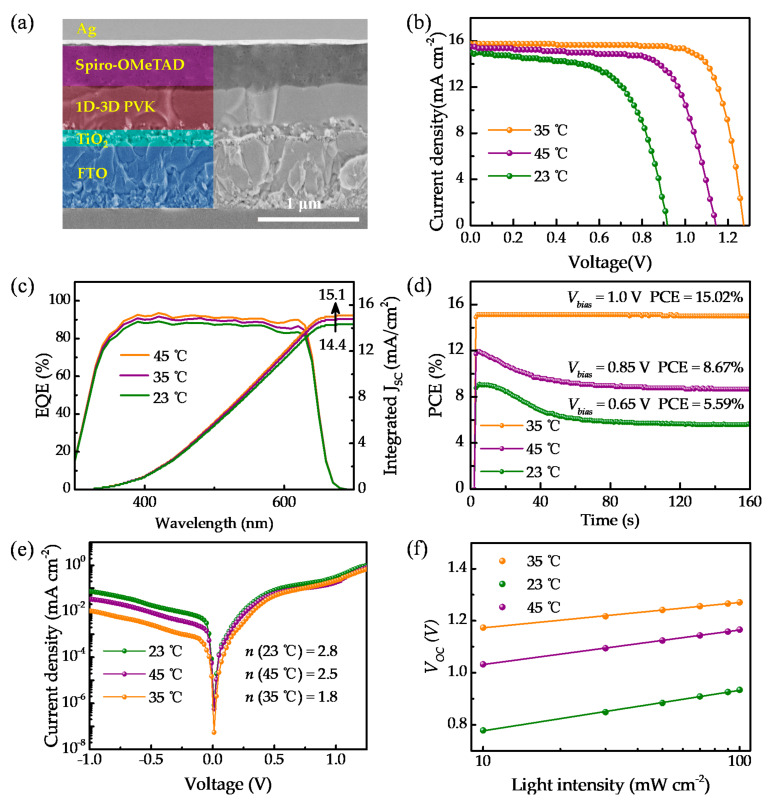
(**a**) The cross-sectional SEM images of optimized PSCs. (**b**) *J–V* curves of the 23 °C, 35 °C, and 45 °C CsPbI_2_Br PSCs under simulated AM 1.5 G illumination (100 mW cm^−2^). (**c**) Corresponding EQE and the integrated photo-current of 23 °C, 35 °C, and 45 °C solar cells. (**d**) Stabilized power output (SPO) as a function of time for the devices fabricated at 23 °C, 35 °C, and 45 °C, respectively. (**e**) Dark *J–V* curves of the 23, 35, and 45 °C CsPbI_2_Br solar cells. (**f**) Plots of light-intensity-dependent *V_OC_* of the 23, 35, and 45 °C CsPbI_2_Br devices.

The cross-sectional SEM image (Figure 7a) of the optimized PSCs shows the functional layer. The thickness of CsPbI_2_Br film (35 °C) is up to 500 nm, which ensures sufficient light absorption. Furthermore, defects in perovskite films are mainly distributed in crystal boundaries [42]. Noticeably, the cross-sectional SEM image reveals that the optimized CsPbI_2_Br film exhibits negligible crystal boundaries. All the excellent microstructure features of the film undoubtedly lead to high-performance optoelectronic properties. Figure 7b provides the *J*–*V* curves of the 23–45 °C CsPbI_2_Br PSCs with the traditional structure (FTO/c-TiO_2_/m-TiO_2_/perovskite/Spiro-OMeTAD/Ag). As shown in Table 1, the best PCE (35 °C) is 15.5%, which is 68% higher than that of the 23 °C device; in addition, the *J*–*V* curves from forward and reverse scanning of the PSCs show hysteresis. Hysteresis is unavoidable in most inorganic PSCs with mixed halide compositions due to the iodide and bromide phase segregation under illumination [43]. Fortunately, the *J*–*V* curve results suggest that the optimized perovskite solar cells have slight hysteresis with respect to the highly crystallized quality. Figure 7c displays the corresponding external quantum efficiency (EQE), and it can be seen that the integrated photocurrent of the optimized CsPbI_2_Br PSC increases from 14.4 to 15.1 mA cm^−2^, compared to the 23 °C device. The corresponding integrated photocurrent is consistent with the measured short-circuit current *J_SC_* value (15.8 mA cm^−2^), and the discrepancy is 4.4%. Figure 7d displays the stabilized power output (SPO) as the function of time for the control and modified solar cells. The best modified device exhibits extremely stable performance with a value as high as 15.02%. In parallel, the devices obtained at 45 and 23 °C yield efficiencies with the values of 8.67% and 5.59%, respectively. The *J*–*V* curves and SPO results suggest that the optimized ambient temperature in perovskite solar cell fabrication is a possible avenue towards a highly efficient and stable perovskite solar cell.

The current density–voltage (*J*–*V*) characteristics of the solar cells at 300 K in the dark are shown in Figure 7e. The lower dark-current density of the optimized device further indicates the reduction of shunt pathways, which may originate from the defective grain boundaries. The dark-current ideality factor (*n*) can be determined from the slope of the exponential regime of dark *J*–*V* characteristics on a semi-logarithmic plot. The dark-current ideality factor of the optimized CsPbI_2_Br PSC decreases from 2.8 to 1.8, as shown in Figure 7e, which means fewer electrically active traps [44,45,46]. The values of *Rs* and *Rsh* of the solar cell can be determined from the voltage dependence of the differential resistance $R_{diff}=\Delta V/\Delta J$ as shown in Table 2 [45,47]. The shunt resistance (*Rsh*) for the optimized device is about 6.8 and 3.9 times that for the 23 and 45 °C devices, respectively. The relative increase in *Rsh* of the optimized PSC could be explained by the better film formation/coverage on the mesoscopic layer compared to the as-prepared films at other ambient temperatures, thus resulting in a reduction in the shunt paths [46,48]. To further evaluate the recombination behavior of the solar cell devices, the dependence of *V_OC_* on light intensities was measured, and the results were plotted as a function of light intensity in logarithm scales as shown in Figure 7f. Ideality factor (*n*) of the devices can be deduced by the slope of *V_OC_* as a function of light intensity according to the following equation [49,50]:

(4) $$V_{OC}=\frac{E_{g}}{q}-\left(\frac{nk_{B}T}{q}\right)In\left(\frac{I_{0}}{I}\right)$$

where $E_{g}$ is the bandgap of light absorber, *K_B_* is the Boltzmann constant, T is the absolute temperature, q is the electron charge, $I_{0}$ is the standard light intensity, and I is the light intensity. Table 2 shows the slopes of 1.6 $k_{B}T/q$ for the optimized device. In parallel, values of 2.2$\;k_{B}T/q$ and 2.6 $k_{B}T/q$ were found for the devices obtained at 45 and 23 °C, respectively. The smaller value of ideality factor implies that trap-assisted Shockley–Read–Hall recombination can be effectively suppressed by adjusting the ambient air temperature to 35 °C, in agreement with the observed increase in *V_OC_* and FF [50].

## 3. Materials and Methods

### 3.1. Materials

The FTO-coated glass (FTO thickness of 2.2 mm, surface resistance of 8 Ω/sq, >85% light transmittance) was purchased from Shang Yang Solar energy (Soochow, China). Lead iodide (purity 99.9%), lead bromide (purity 99%), cesium iodide (purity 99.999%), ethanolamine (purity ≥99.5%), 1,2-dichlorobenzene (purity ≥99%), isopropyl titanate (purity 99.9%), 4-tert-butylpyridine (tBP) (purity 96%), isopropanol (purity ≥99.9%), chlorobenzene (purity 99.5%), ethanol (purity 99.5%), and acetonitrile (anhydrous, purity 99.8%) were purchased from Aladdin (Shanghai, China). Dimethyl sulfoxide (anhydrous, purity ≥99%) was purchased from TCI (Tokyo, Japan). Ni(OCOCH_3_)_2_·4H_2_O (purity 99.9%) was purchased from Macklin (Shanghai, China). Hydrochloric acid was purchased from Guangzhou Chemical Reagent (GuangZhou, China). Bis(trifluoromethylsulfonyl)imide lithium salt, Spiro-OMeTAD (2,2′,7,7′-tetrakis (*N,N*-di-pmethoxyphenylamine)-9,9′-spirobifluorene)) (purity ≥99.8%) and tris(2-(1*H*-pyrazol-1-yl)-4-tert-butylpyridine)cobalt(III) tris(bis-(turfluoromethylsuflonyl)imide) (FK209, Dyesol) were purchased from Xi’an p-OLED (Xi’an, China). 18 NR-T Titania paste was purchased from Greatcell Solar (Queanbeyan, Australia).

### 3.2. Device Fabrication

The inorganic PSCs were fabricated with the structure FTO/c-TiO_2_/m-TiO_2_/CsPbI_2_Br/Spiro-OMeTAD/Ag. The FTO substrates were sequentially cleaned with ethanol, acetone, and isopropanol in an ultrasonic bath for 20 min and dried with nitrogen. To prepare the c-TiO_2_ precursor solution, 700 μL of isopropyl titanate and 70 μL of aqueous hydrochloric acid (2 mol L^−1^) were dissolved into 10 mL anhydrous alcohol. The FTO/glass substrates were treated under UV−ozone for 15 min to form a hydrophilic surface. Then, the 90 μL c-TiO_2_ layers (compact layer) were formed by spin-coating the c-TiO_2_ precursor solution at 2000 rpm for 50 s. After the spin coating, the films were heated on a hotplate at 550 °C for 30 min in air. The heating process included a rise from 50 to 100 °C for 10 min, a 100 °C hold for 10 min, a 10 min rise from 100 to 250 °C, a temperature hold for 10 min, a 10 min rise from 250 to 350 °C, a temperature hold for 10 min, and a rise from 350 to 550 °C. An m-TiO_2_ (mesoporous TiO_2_) solution was prepared by mixing TiO_2_ nanoparticle paste (18 nm diameter) and anhydrous alcohol with the weight ratio of 1:7. The c-TiO_2_/FTO/glass substrates were treated at 200 °C for 20 min. An m-TiO_2_ layer was deposited by spin coating for 60 s at 4000 rpm and then sintered again at 500 °C for 30 min in air. The m-TiO_2_/c-TiO_2_/FTO/glass substrates were treated at 200 °C for 10 min in a dry air glovebox. To prepare a CsPbI_2_Br inorganic perovskite solution, 311.8 mg CsI, 276.6 mg PbI_2_, and 220.2 mg PbBr_2_ were dissolved into 1 mL DMSO to form a 1.2 M solution. After the spin coating with 3000 rpm for 50 s, the film was pre-annealed to brown on a heating plate at 40 °C and then heated at 160 °C for 10 min. Note that we employed a heater and thermal-temperature controller to carefully regulate the temperature. To prepare a Spiro-OMeTAD solution, 100 mg of Spiro-OMeTAD was dissolved in 1 mL of chlorobenzene and mixed with 36 µL tBP, 20 µL Li-TFSI solution (Li-TFSI dissolved in acetonitrile, 516.8 mg mL^−1^), and 12 µL FK209 solution (FK209 in acetonitrile, 375.8 mg mL^−1^). It was spin-coated onto perovskite film at 3000 rpm for 60 s to form a hole transport layer at room temperature. Finally, a 120 nm thick silver layer was thermally evaporated as a top electrode using a shadow mask to form a device active area of 0.09 cm^2^. All the fabrication steps for spin-coating inorganic perovskite films and Spiro-OMeTAD films were performed in a dry air glovebox.

### 3.3. Characterization

The *J–V* performance of the PSCs was analyzed using a Keithley 2400 Source Meter under ambient conditions at room temperature, and the solar simulator of Newport Oriel Sol3A (Newport, Richmond, VA, USA) with simulated AM 1.5 G illumination (100 mW cm^−2^). The scan range was from 1.5 to 0 V. The scan rate was 0.3 V s^−1^. The power output of the lamp was calibrated using a standard Si cell (91, 150 V). The device area of 0.09 cm^2^ was defined by a metal aperture to avoid light scattering from the metal electrode into the device during the measurement. The unsealed CsPbI_2_Br devices were stored in air ambient environments with 15 ± 3% relative humidity for long-term humidity testing. The external quantum efficiency (EQE) of the perovskite solar cell devices was measured by using a spectrum corresponding system of Enlitech QE-R (Enlitech, Kaohsiung City, China), and the light source was a 300 W xenon lamp. The monochromatic light intensity for the EQE measurement was calibrated with a reference silicon photodiode. XRD characterization was performed on an X-ray diffractometer of Bruker D8 Advance with Cu Ka radiation at 40 kV and 40 mA (Bruker, Karlsruhe, Germany). The XPS measurements were performed (Mg anode, 250 W, 14 kV) in an ESCALAB 250Xi (Thermo Electron Corporation, Waltham, MA, USA), and the binding energy of the C1s peak at 284.8 eV was taken as an internal pristine measurement. The surface morphology of films and the cross-sectional images of the PSCs were characterized by scanning electron microscopy (SEM) of Hitachi S-4800 (Hitachi, Tokyo, Japan).

## 4. Conclusions

In summary, we demonstrated the production of efficient inorganic PSCs by using an ambient air temperature assisted crystallization method. The relationship between the ambient temperature inside the glovebox and CsPbI_2_Br crystallization was deeply explored. We found that fixing the temperature at ~35 °C could lead to optimized perovskite films with various advantages, such as large crystalline grains, negligible crystal boundaries, and a pinhole-free surface. Such high-quality thin films exhibited a reduced density of defect states and sufficient enhancement of the carrier extraction efficiency. Consequently, efficient, moisture- and oxygen-tolerant CsPbBrI_2_ PSCs were obtained. The best efficiency of the optimized solar cells was up to 15.5%; the corresponding SPO was 15.02%. Besides, the moisture stability of the device was also enhanced. This work reveals that the optimized ambient temperature is of great significance in the fabrication process of inorganic perovskites, which would be promising to be applied in the future toward scaling up the inorganic PSCs.

## Figures and Tables

**Table 1:** Forward scan and reverse scan performance parameters of perovskite solar cells fabricated at 23 °C, 35 °C, and 45 °C ambient air temperatures.

Device	*J_SC_* (mA cm^−2^)	$V_{OC}\;(V)$	FF	PCE (%)
23 °C-For	14.6 ± 0.3	0.76 ± 0.05	0.51 ± 0.03	5.5 ± 0.8
23 °C-ReV	14.6 ± 0.3	0.88 ± 0.05	0.65 ± 0.03	8.4 ± 0.8
35 °C-For	15.4 ± 0.4	1.21 ± 0.03	0.72 ± 0.02	13.5 ± 0.9
35 °C-Rev	15.4 ± 0.4	1.24 ± 0.03	0.76 ± 0.02	14.5 ± 1.0
45 °C-For	15.0 ± 0.4	1.09 ± 0.06	0.59 ± 0.02	9.7 ± 0.6
45 °C-Rev	15.0 ± 0.4	1.14 ± 0.06	0.68 ± 0.02	11.7 ± 0.6

**Table 2:** Series resistance and shunt resistance extracted from the dark current of solar cell devices prepared at 23 °C, 35 °C, and 45 °C, as well as the *V_OC_* slope at different light intensities.

Device.	*Rs* (Ω cm^−2^)	*Rsh* (Ω cm^−2^)	*V_OC_* Slop (KT/q)
23 °C	2.9	1214	2.6
35 °C	4.2	8484	1.6
45 °C	3.0	2160	2.2

## Data Availability

The data presented in this study is available in article or from the corresponding author.

## Sample Availability

Samples of the compounds produced are available from the authors.
